# Models of housing and support to reduce risks of COVID-19 infection and homelessness: the moving on pilot randomised controlled trial

**DOI:** 10.1186/s40814-025-01718-1

**Published:** 2025-11-04

**Authors:** Peter Mackie, Elizabeth Randell, Calie Dyer, Kim Smallman, Jacqueline Hughes, Robert Trubey, Penelope Farthing, Charlotte Scoble, Guillermo Rodriguez-Guzman, James White, Tim Aubry, Dennis Culhane, Susannah Hume, Kerenza Hood, Faye Greaves, Bethan Pell, Gwenllian Moody, Ligia Teixeira, Victoria Mousteri, Nick Spyropoulos, Rebecca Cannings-John

**Affiliations:** 1 School of Geography and Planning, Cardiff University, Cardiff, Wales; 2 Centre for Trials Research, Cardiff University, Cardiff, Wales; 3 DECIPHer, Cardiff School of Social Sciences, Cardiff University, Cardiff, Wales; 4 School of Psychology & Centre for Research on Educational and Community Services, University of Ottawa, Ottawa, Canada; 5 School of Social Policy & Practice, University of Pennsylvania, Philadelphia, USA; 6 Kings College London, London, England; 7 Centre for Homelessness Impact, London, England; 8 Alma Economics, 43 Tanner St, London, England

**Keywords:** People experiencing homelessness, COVID-19, Local authority, Settled accommodation, Temporary accommodation, Pilot RCT

## Abstract

**Background:**

The UK government ‘Everyone In’ initiative in response to COVID-19 in England saw an unprecedented number of individuals experiencing homelessness moved into temporary accommodation (TA). A limited supply of settled housing meant swift access to settled accommodation (SA) would not be possible for all. This pilot RCT pursued a unique opportunity to examine the feasibility and acceptability of randomising people experiencing homelessness (PEH) to SA or TA and the impact on COVID-19 infection and housing instability.

**Methods:**

A pilot RCT, with embedded process and health economic evaluations. 1:1 participant randomisation to SA (intervention group) or TA (control group). Recruitment in two local authorities (LA) in England. Participants were aged 18 and over, in single-person homeless households, temporarily accommodated by the LA with recourse to public funds. Primary outcomes: (i) LA recruitment; (ii) Participant recruitment; (iii) participant retention; (iv) LA adherence. Secondary outcomes: (i) completeness of data collection at 3 and 6 months; (ii) data linkage: percentage of participants consenting to data linkage and successful match rate.

**Results:**

Of 144 LAs approached, 26 showed interest in participating, two entered the trial. LA hesitancy to participate reflects an unease with trials in services where RCTs are rare. These recruitment challenges resulted in an amendment from full-scale effectiveness RCT to pilot RCT design. Fifty PEH were recruited (29% from 175 approached). Fifty-six percent of participants were retained at 6 months. Fifty percent of randomisation allocations were adhered to by LAs, identifying difficulties in LA systems not amenable to randomisation and a lack of support for randomisation amongst front-line staff. Frontline workers felt strongly that allocations should be based on their judgement. There was a high level of outcome measure completion. All participants consented to sharing identifiers for linkage to health and other data. A match rate with NHS Digital was sought but could not be reported due to procedural challenges.

**Conclusions:**

Whilst not recommended to proceed to a full-scale RCT in its current design, considerable uncertainties remain about the effectiveness and cost effectiveness of different housing interventions on health outcomes, COVID-19 infection and housing stability for PEH.

**Trial registration:**

ISRCTN69564614. Registered on December 16, 2020.

**Supplementary Information:**

The online version contains supplementary material available at 10.1186/s40814-025-01718-1.

## Key messages regarding feasibility

1. Existing uncertainties regarding feasibility: this was one of the first ever pilot randomised controlled trials in the UK with people experiencing homelessness (PEH)
2. Key feasibility findings: the trial provides important new insights into the feasibility and acceptability of randomising participants to Settled Accommodation (SA) or Temporary Accommodation (TA), an important area of research given the absence of trials in this area in the UK. Poor LA recruitment was a major study limitation, as was the low level of LA adherence to randomisation, particularly given LAs were only recruited into the study if they had committed to implement trial processes. The trial showed that telephone interviews with PEH, combined with ongoing communication with Local Authorities (LAs) to ensure up-to-date contact details, alongside relatively small participant incentives, are suitable methods for retaining participants in the study over a 6-month period, albeit with further resources retention might be improved.
3. Implications of findings: LAs are the primary provider of homelessness services in the UK and their participation is key to trial implementation in this field. Recruitment of LAs proved to be particularly challenging. The recruitment of PEH into the study was also difficult. This results from the study`s reliance on busy and under-resourced LAs who were tasked with making initial contact with potential participants, as well as the choices made by PEH. Lessons from recruitment and retention of participants relate to future study design. Engagement in trials may benefit from offers of funding for LAs being coupled with conditions for participation.

## Background

The onset of the COVID-19 pandemic prompted widespread concerns about the potential impact of the virus [1] on the approximately 160,000 households^1^ in Britain experiencing homelessness each year [2]. Fears focused on the potential spread of the virus within communal forms of temporary accommodation, where facilities and air space are shared. These concerns elicited a swift and determined effort to ensure people experiencing street homelessness (PEH) in the UK were safely accommodated in a wide range of emergency accommodation, including hotels, where there would be space to self-isolate and to reduce the risk of transmission of COVID-19 [3]. In England, between March and September 2020, as part of this initial ‘Everyone In’ government response to COVID-19, 10,566 people were living in emergency accommodation and nearly 18,911 people had been moved on to settled accommodation [4]. The Government committed to prevent people from going back to the streets [5] but the limited supply of settled housing meant that swift access to settled accommodation would not be possible for all households.

The original design of this study was to pursue the unique opportunity of a two-arm effectiveness RCT to investigate whether SA prevents COVID-19 infection and reduces housing instability compared to TA (usual care). Due to difficulties in recruiting Local Authorities, the study design was amended to become a pilot study. This pilot RCT examined the feasibility and acceptability of randomising PEH to Settled Accommodation (SA) or Temporary Accommodation (TA) in order to examine whether being housed in SA prevents COVID-19 infection and reduces housing instability for people experiencing homelessness in England. The study had the following primary feasibility objectives, measured using a combination of quantitative and qualitative outcome data to inform a decision to progress to a full-scale trial: (1) feasibility of recruiting local authorities (LAs) and eligible participants; (2) recruitment rates of participants and retention through 3 months and 6 months post-randomisation follow-up data collection; (3) acceptability of the trial and its processes, including randomisation, to single homeless households and LAs and their willingness to participate in a definitive trial. In addition, the following secondary objectives were addressed: (4) adherence to the trial allocation, reach and fidelity (i.e. whether SA was delivered as intended, works as hypothesised, is scalable and sustainable); (5) the feasibility and acceptability of proposed outcome measures for a definitive trial, including resource use and health-related quality of life data, as methods to measure effectiveness of the intervention and to conduct an embedded health economic evaluation within a definitive RCT; (6) feasibility and acceptability of linkage to routinely collected data within a definitive RCT by assessing whether (a) participants were willing to consent for their data to be linked and (b) personal identifiers could be linked to NHS Digital routine datasets.

## Methods

### Aim

To assess the feasibility and acceptability of randomising people experiencing homelessness (PEH) to settled accommodation (SA) or temporary accommodation (TA) and the impact on COVID-19 infection and housing instability.

### Study design

The original design was a parallel two-arm effectiveness RCT to investigate whether SA prevents COVID-19 infection and reduces housing instability compared to TA (usual care) with the aim of recruiting 1200 participants from across six local authorities in England, including London/Metropolitan Boroughs and District/unitary authorities. Due to difficulties in recruiting Local Authorities, the study design was amended to become a pilot study. This resulted in changes to the protocol outcomes and analysis with progression criteria added.

The revised design was a parallel two-arm pilot unblinded RCT with embedded mixed-methods process evaluation and economic analysis. Full methods are detailed by Randell et al. in the peer-reviewed protocol article [6]. A brief description follows.

### Participant selection and randomisation

The trial recruited two LAs in England (one London borough, one south coast). PEH were eligible for the trial if they were aged 18 and over, in a single person homeless household, temporarily accommodated by the LA, had recourse to public funds and were able to provide adequate informed consent to research participation (including competence in English at conversational level or higher). LA staff were trained by the trial team to briefly introduce the study and request consent to pass on potential participant details. The trial team then sought informed consent for study participation. Randomisation was in a 1:1 ratio, stratified (by LA), and constrained by the SA available in LAs. Allocations were communicated to LAs, who then had a 4-week period to return adherence forms to determine compliance with randomised accommodation. For those not offered housing in that time, current housing was recorded.

### Sample size

No formal sample size calculation was performed as the aim of this pilot trial was to assess feasibility rather than effectiveness. The sample size was pragmatically determined based on LAs’ current caseloads, with a target of 50 PEH per LA anticipated within the available timeframe and resources. This approach would allow reasonable precision around key feasibility parameters and aligns with recommendations for pilot studies, where sample size is often guided by practical considerations [7].

### Intervention

Participants were randomised to receive the intervention of SA or to remain in the comparator control condition, TA, provided by LAs. LAs used their standard provision of TA (e.g. hostels), and made offers of settled housing with the relevant level of support in relation to their own assessments for the SA condition. Settled housing could include Private Rented Sector (PRS) (low and medium support), social housing (low and medium support), and housing first (high support). High support was defined as support provided daily, medium support was once per week, and low support less than once a week.

### Outcomes

Primary outcomes were based on progression criteria:

1. LA recruitment: number of LAs recruited as a proportion of those approached and who showed interest.
2. Participant recruitment: percentage of those approached by LAs, and were eligible, consented and thus were willing to be allocated housing according to randomisation.
3. Participant retention: percentage of participants retained at final follow-up as a proportion of those recruited.
4. Adherence: LAs adhering to assignment of participant to randomised allocation.

Secondary outcomes were as follows:

1. Completeness of the following outcomes at 3- and 6-month follow-up
  COVID-19 infection.
  General health (EuroQol-5 Dimension (EQ-5D)) [8].
  Mental health (Generalised Anxiety Disorder assessment (GAD-7) [9] and four measures from the Office for National Statistics (ONS-4)) [10].
  Employment status.
  Income.
  Drug and alcohol use (AUDIT-C) [11].
  Service access use for mental health.
  Drug and alcohol rehabilitation/service use.
  Healthcare service use.
  Cost-effectiveness (including healthcare and mental health service use, and offending).
  Housing stability (the adapted Residential Time Line Follow Back survey) [12].

Data linkage: percentage of participants consenting to data linkage and successful match rate among participants who consented to data linkage.

### Data collection

Participant reported outcome measures were collected via telephone administered questionnaire at baseline, 3 and 6-month follow up. Participants were emailed/posted £20 vouchers as a reimbursement for their time after each questionnaire. Telephone interviews were undertaken by the trial team, ensuring where possible that the same individual contacted the participant at each time point. Contact attempts were staggered so participants were called at various times for increased likelihood of contact.

### Process evaluation

A mixed methods process evaluation drew together quantitative and qualitative data. Blended qualitative methods including qualitative interviews, visual mapping, surveys and narrative reflections were used to gather data from LAs, service users and trial team staff to explore experiences of taking part in the trial, service provision and housing pathways. LAs who did not participate but showed an interest in the study were also interviewed to discover the reasons why they decided not to participate. Fourteen PEH were interviewed from the two participating sites (8 + 6). Nine LA interviews were completed.

### Progression criteria

A traffic light system was used to indicate the feasibility and acceptability of progressing from a pilot to a full-scale trial [6] (see Table 3).

### Data analyses

For each feasibility objective, descriptive statistics are reported as either means and standard deviations or medians and interquartile range (IQR), as appropriate, and categorical data reported as frequencies and proportions. As this was a pilot trial, no hypothesis testing was performed [13]. All statistical analysis was carried out using Stata version 17.

Interviews were transcribed verbatim and analysed using Nvivo 12™. Thematic content analysis of the qualitative data was completed (Braun & Clarke, 2012) [14]. Emergent themes were identified and organised into an analytic framework designed around the process evaluation key domains to explore adherence, acceptability and mechanism(s) of effect—linked to the theories of change.

A cost-effectiveness analysis intended to assess the cost implications of different housing models and outcomes, with a societal perspective and a time horizon comprising the duration of the trial.

### Public involvement

Public involvement was included throughout. Working alongside individuals from the Centre for Homelessness Impact (CHI) with lived experience, all public-facing materials including participant materials were reviewed, and researcher training was developed. One Trial Steering Committee member also had lived experience and helped with interpretation of results.

## Results

### The feasibility of recruiting local authorities and eligible participants

One hundred and forty-four LAs were approached by email by CHI between October 2020 and May 2021. LAs were targeted based on having a large number of PEH accommodated under ‘Everyone In’ and awaiting further move-on (at least 20 PEH). Twenty-six (18%) showed potential study participation with some level of engagement. Of these, 10 LAs had limited engagement (not progressing beyond an initial call or email response with the trial team), and 16 had meaningful engagement (at least one meeting held with the trial team). Two LAs (1.4% of 144 approached; 7.7% of 26 with any engagement) consented to recruit individuals for the study.

**Fig. 1:**
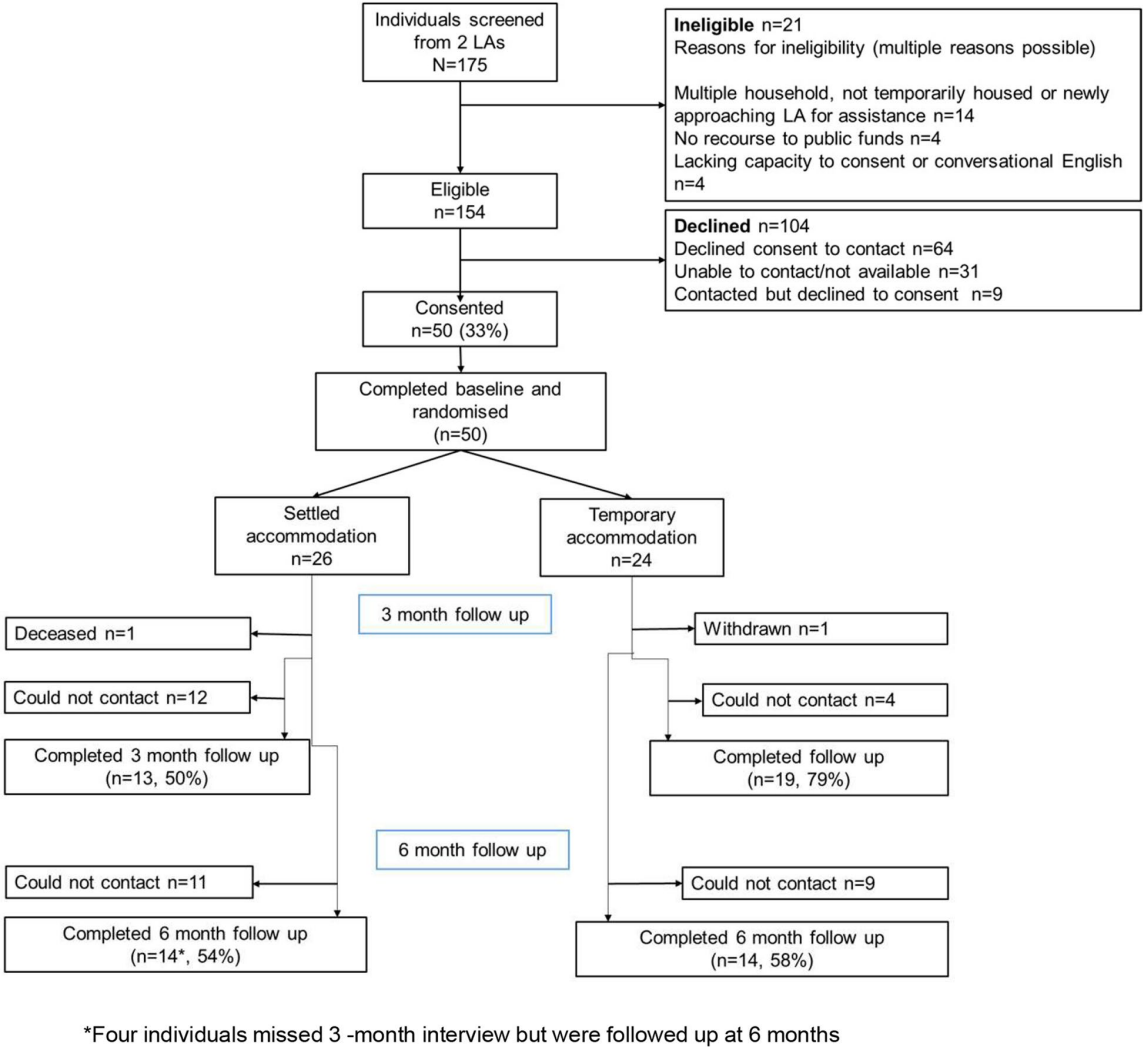
Moving on CONSORT diagram

From the recruited LAs, 175 PEH were screened by LA staff between 2nd February 2021 and 1 st July 2021. 154 (88%) individuals were eligible and of these, 50 (33%) consented, completed the baseline interview, and were randomised to either move on to SA (*n* = 26) or remain in TA (*n* = 24) (Fig. 1). The main reason for ineligibility (*n* = 21) was either living in a multiple household or not being temporarily housed. Eligible individuals mainly declined to participate (*n* = 104) by declining permission for the trial team to make initial contact (*n* = 64, 62%). Participant characteristics were broadly balanced between randomisation arms (Table 1).

**Table 1:** Participant characteristics split by accommodation, as randomised

	*n* (%)	
	Temporary (*n* = 24)	Settled (*n* = 26)
**Characteristics**		
Age (years) (mean, SD)	43.0 (13.5)	44.3 (15.8)
Sex		
Male	14 (60.9)	17 (65.4)
Female	9 (39.1)	9 (34.6)
Not recorded	1	0
Ethnicity		
White	20 (87.0)	20 (76.9)
Black/African/Caribbean	0 (0.0)	4 (15.4)
Mixed/multiple ethnic backgrounds	0 (0.0)	1 (3.8)
Other ethnic group	3 (13.0)	1 (3.8)
Not recorded	1	0
Long-standing physical impairment, illness or disability		
No	7 (29.3)	10 (40.0)
Yes	16 (66.7)	14 (56.0)
Don’t know	1 (4.2)	1 (4.0)
Not recorded	0	1
High-risk of COVID eliciting shielding letter^a^	5 (20.8)	1 (3.8)
High-risk of COVID overall^b^	13 (54.2)	13 (50.0)
Supportive relationship with friends or family	15/23 (65.2)	15/26 (57.7)
Sources of income^c^	***n* = 23**	***n* = 25**
Employed	2 (8.7)	1 (4.0)
Paid work (but not employment)	1 (4.3)	0 (0)
State benefits	21 (91.3)	22 (88.0)
Other	0 (0)	1 (4.0)
Sources of benefit^c^	***n* = 21**	***n* = 22**
Universal credit	16 (76.2)	17 (77.3)
Housing or Council Tax Reduction	2 (9.5)	1 (4.5)
Income support	3 (14.3)	1 (4.5)
Sickness or disability benefits^d^	8 (38.1)	5 (22.7)
Pension benefits (incl. state pension or pension credit)	0 (0)	1 (4.5)
Times in life been homeless (Median, IQR)	1 (1 to 3.75)	2 (1 to 4.25)
Once	15 (62.5)	12 (46.2)
2–9 times	7 (29.1)	9 (34.6)
10 or more	2 (8.3)	5 (19.2)
Accommodation at time of interview		
Hostel	3 (12.5)	4 (15.4)
Emergency accommodation	9 (37.5)	18 (69.2)
Other temporary accommodation^e^	12 (50.0)	4 (15.4)
Smoking status (collected at 3 months interview)	***n* = 19**	***n* = 13**
Never/ex-smoker	6 (31.6)	3 (23.1)
Current smoker (not every day/every day)	13 (68.4)	10 (76.9)

^a^Derived from health conditions questions 1–10 (Supplementary Table S1)

^b^Derived from health conditions questions 1–19 (Supplementary Table S1)

^c^More than one source could be selected

^d^Including Personal Independence Payment or Employment and Support Allowance)

^e^HMO, self-contained, sheltered, temporary supported, supported/social rented housing but meeting conditions of temporary

### Acceptability of the trial and its processes, including recruitment and randomisation, to single homeless households and local authorities and their willingness to participate in a definitive trial

#### People experiencing homelessness

The main motivation to participate amongst PEH was the ability to help others, have their voice heard and to share experiences for others, including researchers and service providers, to learn from. Incentives offered were helpful but not the main motivation of participation. PEH who declined consent to participate could not be interviewed, therefore any problematic trial processes could not be explored with them.

#### Declining local authorities

Many LAs showed interest in participating because they realised the value of evidence in supporting homelessness solutions. The first barrier to LA participation was the inability to fit randomisation into existing processes. For example, LAs could not guarantee a participant access to SA if randomised to that arm because they either had insufficient housing supply, or private landlords could refuse a potential tenant and tenants could refuse an offer. Conversely, LAs claiming to have sufficient housing allocated individuals to SA as soon as they were ‘ready’, meaning randomisation to TA would be an unacceptable denial of accommodation. Importantly, the study was premised on the tenet that there was insufficient SA for all households currently in TA. Second, there were concerns about ceding control over housing allocation, particularly where SA is allocated on the basis of need or length of time on a waiting list. In some instances where allocations into SA were through social housing, barriers were linked specifically to statutory obligations to comply with LA allocations policies. Third, whilst the demands on participating LAs were minimal, existing pressures on LA housing services and limited staff resources, particularly during the COVID-19 pandemic, meant there was little to dedicate to the study in a meaningful way.

#### Participating local authorities

Following detailed discussions, participating LAs accepted all trial processes. However, these proved challenging to implement. Participant recruitment into the study was particularly difficult as overburdened housing staff carrying full caseloads had reduced capacity to explain the study to potential participants. This was often delegated to newer staff with lower caseloads who were unknown to the PEH. The lack of a trusting relationship meant there was some suspicion that participation may be detrimental to their application for housing. These communication challenges were exacerbated by the shift away from in-person engagement during the COVID-19 pandemic. Finally, it also proved challenging to predict availability of SA, nor could firm offers of SA be made in some cases.

### Recruitment rates of participants and retention through 3 months and 6 months post-randomisation follow-up data collection

At 3- and 6-months post-randomisation, 67% (*n* = 32: *n* = 19 TA; *n* = 13 SA) and 58% (*n* = 28: *n* = 14 TA; *n* = 14 SA) respectively, provided follow-up data. 24/50 (48%) were followed up at both 3- and 6-months, 14 (28%) at neither, whilst 8 (16%) were followed up at 3- but not at 6-months. Contact was regained with four participants (8%) at 6-months, who could not be contacted at 3 months.

### Adherence to the trial allocation, reach and fidelity (i.e. whether SA is delivered as intended, works as hypothesised, is scalable and sustainable)

**Fig. 2:**
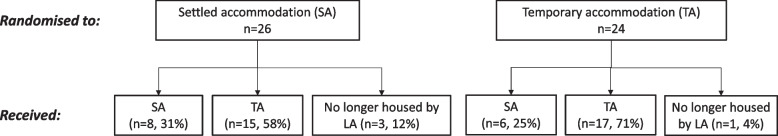
Accommodation randomised to versus received

LAs adhered to randomisation for 25 (50%) out of 50 participants (Fig. 2). From the 26 randomised to receive SA, 8 (31%) were offered SA, 15 (58%) remained in TA, and 3 (12%) were no longer housed by the LA. From the 24 randomised to receive TA, 17 (71%) remained in TA, 6 (25%) were offered SA, and 1 (4%) no longer housed by the LA.

#### Challenges to allocations

Participating LAs often relied on PRS supply from local letting agents. Multiple PEH may be sent to view each property with the landlord deciding who they will accept, in effect a bidding process. LAs therefore sometimes have limited control over who will receive a tenancy. Even where SA was available, LAs sometimes opted to reject randomisation in favour of their own judgements about the most suitable offer for individuals. LAs keen to preserve good relationships with local landlords were hesitant to put some people forward if there was a perceived risk of a tenancy swiftly breaking down.

Participants selected by LAs to receive SA may have been chosen for a reason (e.g. requiring further support, vulnerability) (Table 2). Even though numbers were small, more of those receiving (but not randomised to) SA were female compared to participants randomised to or remain in TA (50% vs 41% respectively), had a long-standing physical impairment, illness or disability (83% vs 59% respectively), had a lower general health score (0.4 vs 0.7 respectively), and a higher AUDIT-C score (19 vs 12.5 respectively).

**Table 2:** Characteristics of those allocated as randomised or not (*n* = 46^*^)

	*n* (%)			
	Allocated as randomised		Allocated not as randomised	
	TA (*n* = 17)	SA (*n* = 8)	Received TA (randomised to SA) (*n* = 15)	Received SA (randomised to TA) (*n* = 6)
Age (years) (mean, SD)	41.9 (14.6)	30.0 (9.8)	49.2 (13.5)	44.3 (11.4)
Male	10/16 (62.5)	6/8 (75.0)	9 (60.0)	3 (50.0)
White ethnic background	15/16 (93.8)	5/8 (62.5)	12 (80.0)	5 (83.3)
Long-standing physical impairment, illness or disability	10/17 (58.8)	3/8 (42.9)	10 (66.7)	5 (83.3)
Supportive relationship with friends or family	11/16 (68.8)	5/8 (62.5)	8 (53.3)	3 (50.0)
Support level assigned by LA				
Low: less than once a week	8 (47.1)	5 (62.5)	10 (66.7)	3 (50.0)
Medium: once a week	9 (52.9)	2 (25.0)	5 (33.3)	11 (16.7)
High: daily	0 (0.0)	1 (12.5)	0 (0.0)	2 (33.3)
Times in life been homeless (Median, IQR)	1 (1 to 2)	1.5 (1 to 7.5)	2 (1 to 4)	2.5 (1 to 5)
Baseline measures				
EuroQoL VAS score^a^ (Median, IQR)	32.5 (30 to 79)	60 (50 to 90)	60 (50 to 75)	42.5 (20 to 70)
EuroQol-5 Dimension (EQ-5D) score^b^ (Median, IQR)	0.7 (0.4 to 0.9)	0.7 (0.4 to 1.0)	0.7 (0.3 to 0.9)	0.4 (0.4 to 0.5)
Generalised Anxiety Disorder assessment (GAD-7)				
Minimal/mild anxiety	7 (41.2)	3/7 (42.9)	7 (46.7)	0 (0.0)
Moderate/severe anxiety	10 (58.8)	4/7 (57.1)	8 (53.3)	6 (100.0)
Used a mental health service	6 (35.3)	4/7 (57.1)	6 (40.0)	2 (40.0)
AUDIT-C^c^ score (Median, IQR)	12.5 (11 to 18)	14 (13.5 to 15.5)	12 (11 to 16)	19 (13 to 23)

^*^Excludes 4 participants that were no longer housed by the local authority

^a^Score range 0–100, higher scores indicate better states of wellbeing

^b^Score range 0–1, higher scores indicate better states of wellbeing

^c^Range from 0 to 20, higher scores indicate higher risk

### The feasibility and acceptability of proposed outcome measures for a definitive trial

While questionnaires were well completed by participants, housing data was found to be imprecise in two ways. First, some had difficulties recalling dates of moving over a 3-month period. Second, distinctions between types of accommodation and definitions of settled and temporary accommodation were not always clear, resulting in some inconsistencies between LA and participant classifications. Interviews took on average 31 min at each time point, with some taking longer due to distress during the call (*n* = 4; 3 at baseline, 1 at 6 months). Concern with adherence to the randomisation and reduced numbers at follow-up, makes an effectiveness assessment of these outcomes uninterpretable. Therefore, summary statistics for each outcome measure, for TA and SA as received (and not necessarily randomised to), are presented in Supplementary Material Table S2.

#### COVID-19 infection

Thirty percent (*n* = 15) of participants reported having had COVID-19 (“Do you think you have had COVID-19?”) at baseline. They reported experiencing fever or fatigue (*n* = 8, 53%), a new continuous cough or muscle ache (*n* = 7, 47%), and shortness of breath, loss of taste, or sore throat (*n* = 6, 40%). At baseline, 66% (*n* = 31/50) reported having been tested for COVID-19, this rose to 91% (*n* = 38/42) at 6 months. Seventy seven percent (*n* = 24/30) reported receiving at least one vaccine by the 6-month follow up (only measured at 3 and 6 months).

#### Housing stability

Of those receiving TA (*n* = 32) and assessed at any point during follow up (*n* = 25), 16 (64%) moved into SA with a median time of 94 days (IQR: 41 to 184 days). Of participants receiving SA (*n* = 14) and assessed at any point during follow up (*n *= 10), 7 (70%) moved into SA with a median time of 66.5 days (IQR: 12 to 80.5 days). Accommodation moves over the study period was only assessed for participants who completed 6-month follow-up interviews (*n* = 24; 17 TA vs. 7 SA). Over 70% (*n* = 5) of participants receiving SA had moved once since baseline (including the initial move from TA to SA), compared to 47% (*n* = 8) allocated to TA. None of those receiving SA had two moves or more (compared to 29% (*n* = 5) of participants allocated to TA).

#### Acceptability of interviews

PEH reported that the interviews were relevant, acceptable, and that researchers carried them out professionally and with respect and compassion. The order and topics of questions resulted in participants giving a comprehensive account of their experiences. For some this realisation and reiteration of circumstances was overwhelming and upsetting. However, others found clarification in the process and some looked forward to their calls, which gave an opportunity to discuss their situation in a safe space.

#### Experiences of researchers on collecting data

Early challenges in data collection processes were quickly resolved, systems of safeguarding and reporting concerns worked well. Researchers noted the emotional demands of engaging with the stories of individuals facing extreme circumstances.

This emotional work was not limited to instances where participants became distressed; rather, it also stemmed from hearing about challenging life experiences, particularly when those experiences were unfamiliar to the researcher (e.g. for those not working in the sector). In addition, listening to participants express visible or audible distress was itself emotionally difficult, regardless of the researcher’s professional background.

### Cost-effectiveness analysis

Similar to the statistical analysis, concern with randomised allocation adherence and small numbers at follow-up meant that a Cost Benefit Analysis (CBA) was not feasible. Instead, an exercise to cost the types of accommodation in one LA was undertaken and will be reported on separately.

### Feasibility and acceptability of linkage to routinely collected data within a definitive RCT by assessing whether (a) participants are willing to consent for their data to be linked and (b) personal identifiers can be linked to NHS Digital routine datasets

All participants consented to personal identifiers being sent to data providers for linkage to health and other data, suggesting that participants were amenable to data sharing. Our intention was to securely send identifiers (forename, surname, date of birth, sex, postcode) to NHS England (was NHS Digital). These would be linked by NHS England to NHS Number using the Master Person Service (MPS). Procedural challenges within NHS England mean that after approximately 2 years of discussions, at the time of writing, we have been unable to commence the linkage process and therefore a match rate cannot be reported. We anticipate that with a high proportion of participants consenting to personal identifiers being sent to data providers, trial participants in this population can be linked to NHS England routine datasets (such as Hospital Episode Statistics or Primary Care datasets) for future projects, even where it is not feasible to collect NHS Number.

### Progression criteria

Results of feasibility parameters and criteria for progression are detailed in Table 3. According to the traffic light system, none of the four progression criteria were met (two red criteria; two amber) and the study should not proceed in its current design to a full-scale RCT.

**Table 3:**
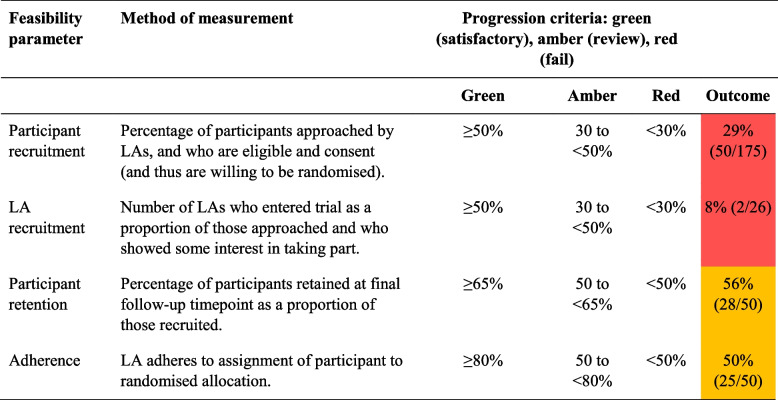
Table of feasibility parameters and criteria for progression to full trial

## Discussion

This study aimed to evaluate the feasibility and acceptability of randomising PEH to SA or TA with the aim of testing whether being housed in SA prevents COVID-19 infection and reduces housing instability. In seeking to situate findings in a wider context and due to the absence of UK-focused trials in this field, comparisons are mostly drawn with North American trials and evaluations with similarly vulnerable populations in the UK.

### Recruitment of local authorities

Rarely have other trials with PEH reported similar hesitancy to participate amongst LAs or service providers. This will in large part reflect an unfamiliarity and unease with trials within UK LA homelessness services where only a small number of RCTs have been conducted and these rarely include random allocation of housing [15]. We uncover several key barriers to LA recruitment, including an inability to fit randomisation into existing LA processes, concerns regarding ceding control over housing allocation, and a lack of staff resources—particularly in the context of COVID-19. Perhaps the most challenging of these to surmount is the fit with existing processes. In these contexts, alternative study designs may be necessary, including natural experiment designs [16]. Where existing processes allow, resource constraints and LA hesitancy could be addressed by offering funding for new services and making participation in the trial a funding condition. For example, in the widely cited At Home Chez Soi Housing First trial in Canada, the evaluation was a key part of the wider demonstration project which received $110 million from the Canadian Government [17, 18], albeit investment of this scale in intervention and evaluation is rare in Canada. In the UK, traditional funders of research are limited to funding evaluations, rather than also supporting the implementation of interventions.

### Recruitment and retention of people experiencing homelessness

The main motivation for trial participation amongst PEH was the possibility to help others and having their voice heard, however participant recruitment was generally difficult. Placing the onus on LA staff, who were busy and often working remotely during the pandemic, was a key barrier. In one of the few other UK trials with a comparable design, similar challenges were observed and only 17 of 1432 potential participants consented to take part. Other trials with PEH (mostly in North America) and with similar populations in the UK (e.g. care experienced young people), often improved participant recruitment by locating additional social workers or similarly skilled staff into services in order to explain the study and either gain consent or refer on to the study team [19–21]. After 6 months, participant retention was also lower than many other North American homelessness trials [12], albeit studies with similar populations in the UK have observed equally low retention rates [22]. Higher retention rates in other studies were achieved through greater investment in proactive retention activities, e.g. offering a participant drop-in [19], and surveillance of electronic sources and physical locations [20]. A particularly effective method involved gaining participant consent for the social services department that administers benefit payments to disclose updated contact details [12].

### Adherence to randomisation

Perhaps the most significant finding was lack of adherence to randomisation by LAs. LAs complied mostly when the allocation aligned with structural housing system constraints and preferences and judgments of LA staff. The pivotal influence of service providers on adherence, including in healthcare settings, has been observed elsewhere [23]. However, in trials where adherence has been high, the trial team generally have a much closer relationship with intervention providers and in some studies it appears the trial team are making the referral directly to the randomised intervention, thereby removing the potential for service providers to circumvent the randomisation process [24].

### Data collection

A paucity of quantitative research and trials on the health and housing outcomes of PEH in the UK [25] means there are few survey tools applied in this context. Completion of a specifically designed survey tool was high and acceptable. Rates of self-reported COVID-19 infection at baseline interview were similar to prevalence rates recorded in several US studies of outbreaks in homeless shelters. Seventy seven percent (*n* = 24/30) of participants reported receiving at least one vaccine by the 6 month follow up. Rates of first vaccination amongst the general population in the UK were approximately 90% by the end of 2021 which appears higher than rates amongst PEH. There were issues in the application of the adapted Residential Time Line Follow Back survey used to capture and assess housing stability. Whilst this tool has been used extensively in a north American context [12], the well-documented [26] opacity in definitions of types of accommodation provided to PEH in the UK (e.g. hostels vs shelters vs supported accommodation) made its application to the UK context challenging. Requesting more details on the accommodation, including an address, and completing post-interview validation of the housing type would address key weaknesses of its implementation in this study.

We recognise that future work would benefit from involving researchers with relevant experience—or lived experience—for a stronger peer research approach. Psychological debriefing proved essential; although support was provided by an experienced team member, input from a trained psychologist would be ideal for managing the emotional demands of this work.

### Data linkage

The study also explored feasibility and acceptability of linkage to routinely collected health data. Unlike North America [27], and some European countries such as Denmark [28], administrative data linkage is a nascent method in UK homelessness research [29]. Encouragingly, all participants consented to their identifiers being sent to data providers for linkage to health and other data. Unfortunately, due to major procedural challenges within NHS Digital, the process of linking to routine data was subject to considerable delay—exceeding 2 years—and could not be completed. This has implications for planning future research in terms of the amount of time allocated to securing NHS Digital data. Assuming data can be matched, this promises the ability to explore health and wider outcomes before and after homelessness and any policy or practice intervention.

### Implications and future research

The key strength of this study was bringing homelessness scholars, trials expertise, LAs, and people who have experienced homelessness, together to undertake one of the first RCTs in the homelessness field in the UK. There is a dearth of robust quantitative evidence on the health, housing, and wider impacts of interventions with PEH in the UK [25] and through this study foundational lessons have been learned for future research. Perhaps most importantly, the implications are for the future design and conduct of trials in this field.

Guided by the Medical Research Council (MRC) framework for developing and evaluating complex interventions [30], we recognise that this study primarily tested the feasibility of the evaluation design—particularly the implementation of an RCT within a complex and resource-constrained public service context—rather than the feasibility of the intervention (Settled Accommodation) itself. Future studies would benefit from making this distinction explicit from the outset. Furthermore, this was a post-implementation evaluation which presents particular challenges in applying experimental designs where services are already embedded and allocation procedures are established. As per the MRC framework, the next steps could include conducting an Evaluability Assessment [31] to systematically assess readiness for full-scale evaluation and refine intervention theory, processes, and outcomes.

A minority of progression criteria were met, therefore the study is not recommended to proceed in its current design to a full scale RCT. Additionally, the unique context provided by the COVID-19 pandemic has passed. However, there remain considerable uncertainties about the effectiveness and cost effectiveness of different types of housing intervention on health outcomes (including COVID-19 infection) and housing stability. It is anticipated that the findings from this study will help shape the funding, design and implementation of future UK-based trials with PEH.

## Conclusion

Whilst this study is not recommended to proceed in its current design to a full-scale RCT, considerable uncertainties remain about the effectiveness and cost effectiveness of different types of housing intervention on health outcomes and housing stability for PEH. However, this pilot trial, positioned as one of the first RCTs in the UK homelessness field provides foundational lessons for future research.

In addition to specific lessons on the recruitment and retention of study participants, and the suitability of research tools, the crucial lesson relates to the study design and ability to evaluate of complex interventions. In some cases, existing LA processes may render randomisation with perfect adherence implausible, in which case other designs such as ‘encouragement designs’ or natural experiments may be necessary. Yet, in other contexts, where the barriers to randomisation are surmountable and centre on resource limitations and operational concerns, alternative approaches need to be explored to engage with organisations taking part. In particular, UK funders should consider taking the rare step of supporting both intervention implementation and evaluation concurrently, making trial participation a condition of receipt of new service funds. Situating future research within the MRC framework offers a systematic approach to refining evaluation design, developing robust programme theory, and ensuring policy relevance and feasibility.

Despite progression criteria not being met for the current design, ongoing evaluation should explore potential refinements to RCT designs or iterative re-designs to overcome identified challenges, rather than ruling out all future RCTs. The lessons from this pilot are vital for shaping the funding, design, and implementation of robust future UK-based trials in this critical field.

## Supplementary Information

Supplementary Material 1: Table S1. Proposed participant-reported outcome measures by trial arm for all participants.

## Data Availability

De-identified quantitative and qualitative datasets have been deposited in the UK Data Service (DOI: https://doi.org/10.5255/UKDA-SN-855729).

## Abbreviations

CBA: Cost benefit analysis
CHI: Centre for Homelessness Impact
CTR: Centre for Trials Research
DMC: Data Monitoring Committee
EQ5D: EuroQol-5 Dimension
GAD-7: Generalised Anxiety Disorder Assessment
IQR: Interquartile Range
LA: Local authority
MHCLG: Ministry of Housing, Communities and Local Government
MRC: Medical Research Council
NHS: National Health Research
ONS-4: Office for National Statistics
PEH: Person Experiencing Homelessness
PRS: Private rented sector
RCT: Randomised controlled trial
RSL: Registered social landlord
SA: Settled accommodation
SPIRIT: Standard Protocol Items: Recommendations for Interventional Trials
TA: Temporary accommodation
TMG: Trial Management Group
TSC: Trial Steering Committee
UK: United Kingdom

## Footnotes

1 In terms of this statistic, a household refers to not just the individual who is homeless but also anyone who normally lives with them. This derives from homelessness legislation which entitles households—rather than only individuals—to support.
