# Supplementary material for: Models of housing and support to reduce risks of COVID-19 infection and homelessness: the moving on pilot randomised controlled trial
Source: Pilot Feasibility Stud. 2025 Nov 4;11:132. doi: 10.1186/s40814-025-01718-1 (PMC12584515; doi:10.1186/s40814-025-01718-1)
Supplement: Supplementary file 1 — Supplementary Material 1: Table S1. Proposed participant-reported outcome measures by trial arm for all participants. [file 40814_2025_1718_MOESM1_ESM.docx]

# Supplementary Material

**Table 1. COVID Risk health conditions.**

|  | **Health condition** |
| --- | --- |
| 1 | Have you ever had an organ transplant |
| 2 | Are you currently receiving chemotherapy or antibody treatment for cancer, including immunotherapy |
| 3 | Are you currently receiving intense radiotherapy for lung cancer |
| 4 | Are you currently having cancer treatment that affects the immune system (e.g. protein kinase inhibitors or PARP inhibitors) |
| 5 | Do you currently have blood or bone marrow cancer? (leukaemia, lymphoma, myeloma) |
| 6 | Have you had a bone marrow or stem cell transplant in the last 6 months |
| 7 | Are you currently taking immunosuppressant medication? |
| 8 | Have you been told by a doctor you have a severe lung condition (e.g. cystic fibrosis, severe asthma or severe COPD) |
| 9 | Do you have any condition associated with a very high risk of getting infections (e.g. sickle cell or SCID) |
| 10 | Are you taking any medication that makes you more vulnerable to infections (high doses of steroids or immunosuppressant medicines) |
| 11 | Do you have a lung condition that is not severe (e.g. asthma, COPD, emphysema or bronchitis) |
| 12 | Do you have heart disease |
| 13 | Do you have diabetes |
| 14 | Do you have chronic kidney disease |
| 15 | Do you have liver disease (e.g. hepatitis) |
| 16 | Do you have a condition affecting the brain or nerves (e.g. Parkinson’s disease, motor neurone disease, multiple sclerosis or cerebal palsy) |
| 17 | Do you have a condition meaning there is a high risk of getting infections |
| 18 | Are you taking medication that can affect the immune system (e.g. low doses of steroids) |
| 19 | Do you have a BMI 40 or above |

Table 2. Proposed participant-reported outcome measures by trial arm for all participants

| **Measure** | **TA as randomised** |  | **SA as randomised** |  | |
| --- | --- | --- | --- | --- | --- |
|  | **Baseline 3 months** | **6 months** | **Baseline** | **3 months** | **6**  **months** |
|  | **n=24 n=19** | **n=14** | **n=26** | **n=13** | **n=14** |
| **COVID-19** |  |  |  |  |  |
| Self-reported having COVID-19* | n (%) 7 (29.2) 1 (5.3)  *missing* | 3 (21.4) | 8 (30.8) | 2 (15.4) | 0 (0.0) |
| Tested for COVID-19* | n (%) 18 (75.0) 12 (63.2)  *missing* | 10 (71.4) | 13 (50.0) | 7 (53.8) | 8 (57.1) |
| Tested positive (of those tested)* | n (%) 3 (16.7) 0 (0.0)  *missing* | 1 (10.0) | 2 (15.4) | 0 (0.0) | 0 (0.0) |
| Received a vaccine for COVID- | *-* 11 (57.9) | 10 (71.4) | *-* | 8 (61.5) | 10 |
| 19* | n (%) |  |  |  | (71.4) |
|  | *missing -* *0* | *0* | *-* | *0* | *0* |
| Received COVID-19 vaccine by 6 | n (%) 12/16 (75.0) |  | 12/15 (80.0) |  |  |
| months | *missing* *8* |  | *11* |  |  |
| **General and Mental Health** |  |  |  |  |  |
| EuroQol-5, score range 0-100, | Median (IQR) 0.72 (0.54, 0.90) | 0.77 (0.30, 1.00) | 0.74 (0.42, 0.94) | 0.62 (0.42, 0.94) | 0.75 |
| higher scores indicate better |  |  |  |  | (0.33, |
| states of wellbeing | *missing* |  |  |  | 1.00) |
| EuroQol-5 VAS score, score | Median (IQR) 55 (48.75, 75) 60 (18.75, 76.25) | 35 (25, 75) | 70 (45, 72.5) | 62.5 (33.75, 81.25) | 70 (50, |
| range 0-100, higher scores  indicate better states of wellbeing |  |  |  |  | 77.5) |

|  | **Baseline** | **3 months** | **6 months** | **Baseline** | **3 months** | **months** |
| --- | --- | --- | --- | --- | --- | --- |
|  | **n=24** | **n=19** | **n=14** | **n=26** | **n=13** | **n=14** |
| Generalised Anxiety Disorder Median (IQR) | 15 (4.5, 19) | 12 (2.5, 17.5) | 8.5 (2.5, 15.5) | 13 (1.5, 17.5) | 5 (1, 14.75) | 18 (3.5, |
| (GAD-7), score ranges from 0 to |  |  |  |  |  | 21) |
| 21, higher scores indicate severe |  |  |  |  |  |  |
| anxiety |  |  |  |  |  |  |
| Minimal anxiety n (%) | 6 (25) | 6 (35.3) | 5 (35.7) | 9 (36.0) | 6 (50.0) | 4 (30.8) |
| Mild anxiety n (%) | 2 (8.3) | 1 (5.9) | 2 (14.3) | 2 (8.0) | 1 (8.3) | 0 (0.0) |
| Moderate anxiety n (%) | 3 (12.5) | 4 (23.5) | 2 (14.3) | 4 (16.0) | 2 (16.7) | 1 (7.7) |
| Severe anxiety n (%) | 13 (54.2) | 6 (35.3) | 5 (35.7) | 10 (40.0) | 3 (25.0) | 8 (61.5) |

Office for National Statistics (ONS)-4, score range 0 (not at all) to 10 (completely), higher scores indicate better outcomes

| Overall how happy with your | Median (IQR) | 2 (0, 5.75) | 5 (2, 8) | 7 (2.75, 8.25) | 5 (3.5, 7) | 6 (4, 7.5) | 4 (2.5, |
| --- | --- | --- | --- | --- | --- | --- | --- |
| life are you nowadays | 7.5) | | | | | | |
| Overall, to what extent do | Median (IQR) | 1.5 (0, 8) | 5.5 (2.75, 8) | 8 (2, 10) | 6 (2, 7) | 7 (6, 9.5) | 6 (3, 9) |

you feel that the things you do in your life are worthwhile?

| Overall, how happy did you  yesterday? | Median (IQR) | 2.5 (0 to 5.25) | 6 (2, 8) | 7 (4.75, 8.25) | 5 (3.5, 8) | 7 (3.5, 8.5) | 6 (3,  7.5) |
| --- | --- | --- | --- | --- | --- | --- | --- |
| Overall, how anxious did you yesterday? | Median (IQR) | 3 (0, 7.5) | 5.5 (2, 8) | 3.5 (0, 9) | 3.5 (0, 5.75) | 6 (1, 8.5) | 7 (1,  10) |

feel

feel

|  | **Baseline** | **3 months** | **6 months** | **Baseline** | **3 months** | **months** |
| --- | --- | --- | --- | --- | --- | --- |
|  | **n=24** | **n=19** | **n=14** | **n=26** | **n=13** | **n=14** |
| **Income and employment** |  |  |  |  |  |  |
| Employed* n (%) | 2 (8.7) | 2 (10.5) | 1 (7.1) | 1 (4.0) | 2 (15.4) | 0 (0.0) |
| State benefits* n (%) | 21 (91.3) | 16 (84.2) | 13 (92.9) | 22 (88.0) | 10 (76.9) | 13 |
|  |  |  |  |  |  | (92.9) |
| Paid work but not employment* n (%) | 1 (4.3) | 1 (5.3) | 1 (7.1) | 0 (0.0) | 0 (0.0) | 1 (7.1) |
| **individuals can select more than missing* | *1* | *0* | *0* | *1* | *0* | *0* |
| *one option* |  |  |  |  |  |  |
| **Alcohol and substance misuse** |  |  |  |  |  |  |
| Current illegal drug use, new n (%) | 2 (8.3) | 2 (11.1) | 3 (21.4) | 9 (34.6) | 4 (30.8) | 2 (14.3) |
| psychoactive substances or |  |  |  |  |  |  |
| misuse of prescription medication |  |  |  |  |  |  |
| *missing* | *0* | *1* | *0* | *0* | *0* | *0* |
| AUDIT-C, score range from 0-20, Median (IQR) | 13 (11, 19) | 14 (12.5, 17) | 13.5 (11, 21) | 14 (11, 16.25) | 11 (11, 13) | 12 (11, |
| higher scores indicate higher risk |  |  |  |  |  | 16) |
| *missing* | 1 | 2 | 0 | 0 | 0 | 0 |
| **Service Use** |  |  |  |  |  |  |
| Service use for Mental health in n (%) | 8 (34.8) | 6 (35.3) | 6 (42.9) | 10 (40.0) | 4 (30.8) | 4 (30.8) |
| past 3 months (baseline is past 12 |  |  |  |  |  |  |
| months), includes therapists, but |  |  |  |  |  |  |
| not advice or help from a GP |  |  |  |  |  |  |
| *missing* | *1* | *2* | *0* | *1* | *0* | *1* |

|  | **Baseline** | **3 months** | **6 months** | **Baseline** | **3 months** | **months** |
| --- | --- | --- | --- | --- | --- | --- |
|  | **n=24** | **n=19** | **n=14** | **n=26** | **n=13** | **n=14** |
| Drug misuse - service use n (%) | 4 (16.7) | 3 (16.7) | 2 (14.3) | 9 (34.6) | 3 (23.1) | 0 (0.0) |
| *missing* | *0* | *1* | *0* | *0* | *0* | *0* |
| Alcohol support service use n (%) | 6 (26.1) | 2 (11.1) | 3 (21.4) | 4 (15.4) | 2 (15.4) | 0 (0.0) |
| *missing* | *1* | *1* | *0* | *0* | *0* | *0* |
| GP or Nurse Walk-In centre or n (%) | 18 (75.0) | 14 (77.8) | 11 (78.6) | 19 (72.0) | 8 (61.5) | 12 |
| service (This can include at day centres, hostels or other services)  *missing* | *0* | *1* | *0* | *1* | *0* | (85.7)  *0* |
| Accident and Emergency (A&E) n (%) services for an illness or injury  *missing* | 13 (54.2)  *0* | 5 (27.8)  *1* | 11 (44.0)  *0* | 11 (44.0)  *1* | 2 (15.4)  *0* | 3 (21.4)  *0* |
| Ambulance use n (%) | 12 (50.0) | 3 (16.7) | 5 (35.7) | 9 (36.0) | 0 (0.0) | 2 (14.3) |
| *missing* | *0* | *1* | *0* | *1* | *0* | *0* |
| Hospital n (%) | 12 (50.0) | 5 (27.8) | 6 (42.9) | 9 (36.0) | 5 (38.5) | 5 (35.7) |
| *missing* | *0* | *1* | *0* | *1* | *0* | *0* |
